# Supplementary material for: Utility of VIDAS® Dengue Diagnostic Assays to Differentiate Primary and Secondary Dengue Infection: A Cross-Sectional Study in a Military Hospital from Colombia
Source: Trop Med Infect Dis. 2025 Jan 29;10(2):40. doi: 10.3390/tropicalmed10020040 (PMC11860576; doi:10.3390/tropicalmed10020040)
Supplement: Supplementary file 1 [file tropicalmed-10-00040-s001.zip › tropicalmed-3372851-supplementary.pdf]

Supplementary Materials

First, data of patients in the “primary DENV infection without warning signs” category (42 patients) were compared with those in the “primary DENV infection with warning signs” group (34 patients), analyzing IgM, IgG, and the IgG/IgM ratio values.

The initial step was to validate whether the data followed a normal distribution using the Shapiro-Wilk test. This statistical test assesses whether a dataset conforms to a normal distribution. It is particularly useful for small to medium-sized samples. If the p-value is less than the significance level of 0.05, the null hypothesis of normality is rejected.

Table S1 presents key statistical parameters for each group, along with the corresponding p-values. These results demonstrate that the groups do not follow a normal distribution.

Table S1. Statistical Summary for the Groups " primary DENV infection without warning signs" and " primary DENV infection with warning signs" Regarding IgM Values

GROUP	Mean (IgM)	Median (IgM)	Standard Deviation (IgM)	Min (IgM)	Max (IgM)	p (Shapiro-Wilk)-(IgM)
**PRIMARY DENV INFECTION WITHOUT WARNING SIGNS**	11.33	1	22,87	0	127	0.00055
**PRIMARY DENV INFECTION WITH WARNING SIGNS**	16.26	4	22,6	0	79	0.044

Since the groups did not follow a normal distribution, the Mann-Whitney U test was applied. Also known as the Mann-Whitney-Wilcoxon test or the Wilcoxon rank-sum test, this non-parametric statistical method compares two independent groups to determine whether significant differences exist in their distributions.

When comparing the "primary DENV infection without warning signs" group with the "primary DENV infection with warning signs" group for IgM values using the Mann-Whitney U test, a p-value of 0.1847 was obtained. This result indicated that there were no statistically significant

differences between the groups. The analysis conducted for IgM values in these two groups was also applied to the IgG values and the IgG/IgM ratio.

Tables S2 and S3 present the summary statistics for IgG and IgG/IgM, respectively, demonstrating that these variables also do not follow a normal distribution based on their p-values. Therefore, the Mann-Whitney U test was also used for these comparisons.

Table S4 summarizes the p-values for the comparisons between the groups for IgM, IgG, and the IgG/IgM ratio. Consistently, no statistically significant differences were observed for any of these variables across the groups.

Table S2. Summary statistics for IgG values in the "primary DENV infection without warning signs" and "primary DENV infection with warning signs"

GROUP	Mean (IgG)	Median (IgG)	Standard Deviation (IgG)	Min (IgG)	Max (IgG)	p (Shapiro-Wilk)-(IgG)
**PRIMARY DENV INFECTION WITHOUT WARNING SIGNS**	1.47	0	6,08	0	39	0.00000000000024
**PRIMARY DENV INFECTION WITH WARNING SIGNS**	1.44	0	3,79	0	16	0.00000000029

Table S3. Summary Statistics for IgG/IgM Ratios in the "primary DENV infection without warning signs" and "primary DENV infection with warning signs"

GROUP	Mean (IgG/IgM)	Median (IgG/IgM)	Standard Deviation (IgG/IgM)	Min (IgG/IgM)	Max (IgG/IgM)	p (Shapiro-Wilk)-(IgG/IgM)
**PRIMARY DENV INFECTION WITHOUT WARNING SIGNS**	0.10	0	0.22	0	1	0.00055
**PRIMARY DENV INFECTION WITH WARNING SIGNS**	0.06	0	0.17	0	0.7619	0.044

Table S4. Results of the Mann-Whitney U Test for IgM, IgG, and IgG/IgM Ratios in the "primary DENV infection without warning signs" and "primary DENV infection with warning signs"

GROUP	p- value (IgM)	p- value (IgG)	p- value (IgG/IgM)
**PRIMARY DENV INFECTION WITHOUT WARNING SIGNS**	0.18	0.66	0.80
**PRIMARY DENV INFECTION WITH WARNING SIGNS**			

The process described for comparing the “primary DENV infection without warning signs” and “primary DENV infection with warning signs” groups was repeated for two additional comparisons: “secondary DENV infection without warning signs” (31 patients) versus “secondary DENV infection with warning signs” (34 patients), and “secondary DENV infection (NS1 positive)” (70 patients) versus “secondary DENV infection (NS1 negative). The same statistical methodology was employed.

Table S5 provides descriptive statistics for the groups, while **Table S6** summarizes the results of the Mann-Whitney U test applied to these comparisons.

Table S5. Results of the Mann-Whitney U Test for the Comparison of Groups

PARAMETER	SECONDARY DENV INFECTION WITHOUT WARNING SIGNS	SECONDARY DENV INFECTION WITH WARNING SIGNS	SECONDARY DENV INFECTION (NS1 POSITIVE)	POSSIBLE SECONDARY DENV INFECTION (NS1 NEGATIVE)
Mean (IgG)	20.58	39.26	30.528571	57.07
Standard deviation (IgG)	25.15	29.78	29.013601	20.50
Median (IgG)	10	49	15.5	59.5
Min (IgG)	1	1	1	6
Max (IgG)	78	82	82	82
p (Shapiro-Wilk) (IgG)	0.0000032	0.00029	0.000000061	0.0005172
Mean (IgM)	1.32	1.64	1.54	5.78
Standard deviation (IgM)	2.31	2.92	2.71	10.31
Median (IgM)	0	0	0	2
Min (IgM)	0	0	0	0
Max (IgM)	9	13	13	49
p (Shapiro-Wilk) (IgM)	0.00000021	0.0000000557	0.0000000000080	0.0000000012
Mean (IgM/ IgG)	11.49	21.82	17.65	18.89
Standard deviation (IgM/ IgG)	12.27	19.25	16.9	14.6
Median (IgM/ IgG)	6.4	16.4	12	17.33
Min (IgM/ IgG)	1.22	2	1.22	1.4
Max (IgM/ IgG)	39	75	75	54
p (Shapiro-Wilk) (IgM/ IgG)	0.01751	0.0158	0.00055	0.0440

Table S6. Results of the Mann-Whitney U Test for the Comparison of Groups

GROUP	P - value (IgM)	P- value (IgG)	P value (IgG/IgM)
**SECONDARY DENV INFECTION WITHOUT WARNING SIGNS**	0.6	0.026	0.0690
**SECONDARY DENV INFECTION WITH WARNING SIGNS**			
**SECONDARY DENV INFECTION (NS1 POSITIVE)**	0.00112	0.0000058	0.560
**POSSIBLE SECONDARY DENV INFECTION (NS1 NEGATIVE)**			

All hypothesis tests and calculations were performed using Python version 3.10.12 with the stats library.
